# Economic Analysis of a Rest–Shade–Hydration–Sanitation Program at a Nicaraguan Sugar Mill

**DOI:** 10.5334/aogh.4753

**Published:** 2025-11-08

**Authors:** Zachary J. Schlader, Thomas Boswell, Heath Prince, Catarina Wesseling, Fabiano A. Amorim, Dinesh Neupane, Esteban Arias, Scarlette Poveda, Erik Hansson, Rebekah A.I. Lucas, Kristina Jakobsson, David H. Wegman, Jason Glaser

**Affiliations:** 1 La Isla Network, Washington D.C., USA; 2 Department of Kinesiology, Indiana University School of Public Health – Bloomington, Bloomington, IN, USA; 3 Lyndon B. Johnson School of Public Affairs, The University of Texas at Austin, Austin, TX, USA; 4 Department of Health, Exercise, and Sports Sciences, University of New Mexico, Albuquerque, NM, USA; 5 Department of International Health, Bloomberg School of Public Health, Johns Hopkins University, MD, USA; 6 Occupational and Environmental Medicine, School of Public Health and Community Medicine, Sahlgrenska Academy, University of Gothenburg, Gothenburg, Sweden; 7 School of Sport, Exercise and Rehabilitation Sciences, University of Birmingham, Birmingham, United Kingdom; 8 Department of Public Health, University of Massachusetts Lowell, Lowell, MA, USA

**Keywords:** occupational heat stress, sugarcane, return on investment, intervention, productivity

## Abstract

*Background:* Occupational heat stress mediated acute kidney injury (AKI) has been linked to the development of chronic kidney disease of non-traditional causes (CKDnt) in agriculture workers. Rest–shade–hydration–sanitation (RSH-S) programs are promising interventions for preventing CKDnt. An obstacle to the implementation of RSH-S programs is the concern that the reduced work time associated with these programs may reduce productivity and earnings.

*Objective:* This study analyzes the economic impact of an RSH-S program implemented at a sugar mill in Nicaragua.

*Approach:* Data were obtained from the sugar mill over a six-year, five-harvest period (Harvest 1: 2017–2018 through Harvest 5: 2021–2022). Data included health and productivity metrics and RSH-S program costs. During Harvest 1, existing heat mitigation strategies were in place but were not optimal. Thus, 2017 was considered the pre-RSH-S (baseline) period. Over subsequent harvests, progressively improved RSH-S programs were implemented. A cost-benefit analysis was conducted to estimate the return on investment of the RSH-S program. The analysis considered both fixed and variable costs associated with the program. Benefits were calculated based on productivity improvements and reductions in AKI cases.

*Findings:* As soon as 2020, the costs of implementing the RSH-S program were offset by savings resulting from increased productivity and reduced cases of AKI. The RSH-S program yielded a positive return on investment from 2020 and onward. The average return on investment over the five-year period was 0.02 (or a return of $1.02 for every $1.00 invested), which takes into consideration the first two years of the intervention in which there was a negative return on investment. In 2022, every $1.00 invested in the RSH-S program saw a return of $1.60.

*Conclusions:* Implementing an RSH-S program can provide both economic and health benefits, particularly in locations where climate change is increasing the already present risk and burden of occupational heat stress.

## Background

The most evident manifestation of climate change is the heightened intensity, frequency, and duration of heat exposure [1]. Less well recognized are the risks posed by this heat exposure when combined with the physical activity required by many occupational tasks, which renders outdoor workers uniquely and disproportionately vulnerable to the health impacts of climate change [2]. Seemingly occurring in parallel with climate change, epidemics of chronic kidney disease not related to traditional causes of diabetes, hypertension, obesity, or chronic kidney disease of non-traditional causes (CKDnt) have emerged globally, including in Mesoamerica [3, 4]. A leading hypothesis is that occupational heat stress, a function of both environmental heat exposure and intense manual labor [5], catalyzes the development of CKDnt following single or repeated episodes of heat-induced acute kidney injury (AKI) [6]. Thus, CKDnt is arguably the first occupational disease recognized to be caused by climate change [7]. This occupational heat stress mediated AKI-to-CKDnt hypothesis contends that elevations in internal (core) body temperature increase the risk of developing AKI, which is worsened in the presence of dehydration [5]. This hypothesis is supported by (i) a high CKD mortality in areas where excessive occupational heat stress is common [8]; (ii) clinical data demonstrating that AKI caused by heat stroke increases the likelihood of developing CKD [9]; (iii) data from rodents demonstrating that CKD can be caused by repetitive bouts of heat-induced AKI [10, 11]; (iv) positive associations between AKI and the diagnosis of CKDnt in sugarcane workers [12]; and (v) the introduction of a rest–shade–hydration–sanitation (RSH-S) program that reduces dangerously high core temperatures, reduces the incidence of AKI, and attenuates the fall in kidney function across the harvest in sugarcane cutters [13–15]. To this latter point, despite data supporting the beneficial renal health effects of an RSH-S program, a major obstacle for RSH-S program success is that both employers and workers often worry that reduced work time with the RSH-S program will decrease productivity and earnings.

It is well established that occupational heat stress is a major economic burden. For example, occupational heat stress accounts for an estimated >650 billion lost labor hours annually worldwide, comparable to those caused by the COVID-19 pandemic [16], and this burden is expected to worsen with climate change [17]. However, estimates of the financial benefits of implementing RSH-S programs to counter the impacts of occupational heat stress are preliminary to date. Indeed, initial estimates indicate that worker productivity (e.g., tons of sugarcane cut) decreases with increased heat stress, but that implementation of RSH-S reverses these decrements such that, despite reductions in functional working time, productivity is maintained [18]. Thus, RSH-S programs likely have positive economic consequences. That said, aside from preliminary analyses conducted by our group [19, 20], a comprehensive economic assessment of the potential benefits of an RSH-S program incorporating adverse health outcomes (specifically AKI), productivity, and RSH-S program fixed and annual costs has not been undertaken.

This case study analyzes the economic impact of an RSH-S program, the Adelante Initiative, implemented at the Ingenio San Antonio (ISA) sugar mill in Chichigalpa, Nicaragua (https://adelanteinitiative.org/). Sugar is one of the most important crops cultivated in Nicaragua, accounting for over 4% of the Gross National Product [21]. Kidney disease is the second leading cause of death in Nicaragua [22]. Chichigalpa is in a region with one of the highest known rates of CKDnt in the world, even compared to other Mesoamerican regions or countries, likely due to the combination of intense sugarcane cultivation and hot and humid weather [8]. Indeed, the weather in Chichigalpa is consistently hot and humid [8], and the frequency and severity of extreme heat in the region are predicted to increase due to climate change. ISA is one of the largest producers of sugar in Nicaragua [23], with complex production processes, including both manual and mechanized harvesting. This case study focuses on manual harvesting, which, despite mechanization, is and will remain a requirement at ISA due to the terrain, sugarcane, and other production conditions.

With this background, the purpose of this case study is to provide an economic assessment of the implementation of an RSH-S program at ISA. This cost-benefit analysis incorporated costs related to AKI, lost productivity, and absenteeism, alongside the fixed and annual costs associated with the implementation of the RSH-S program. Using this information, we calculated and report the return on investment (ROI) for the RSH-S program over five harvest periods.

## Approach

The human subjects aspects of this case study were approved by the Comité de Ética para Investigaciones Biomédicas (CEIB), Facultad de Ciencias Médicas, Universidad Nacional Autónoma de Nicaragua (UNAN- León), FWA000045231/IRB00003342 and the study was carried out in accordance with the Declaration of Helsinki, except for registration in a database. In all instances, trained staff apprised all workers of the study objectives and procedures and answered any questions before participants signed an informed consent.

This case study presents an economic assessment of the RSH-S program implemented for cane cutters (both those cutting burned cane and those cutting cane for seed [seed cutters]) at ISA as assessed over five harvest periods, with data presented from 2017 to 2018 (Harvest 1) through 2021–2022 (Harvest 5). Data included health and productivity metrics and RSH-S program fixed and annual costs (described in detail below). The initial observations, development, and implementation of the RSH-S program at ISA have been described previously in detail [19, 24]. Briefly, existing heat mitigation strategies were examined among sugarcane workers during Harvest 1 of the Adelante Initiative. During the harvest, existing heat mitigation strategies were in place but were not considered to be ideal. For example, water and shade were available but these resources were not convenient for workers to access [19]. Thus, for the purposes of this case study, 2017–2018 is considered the pre-RSH-S (baseline) period (Harvest 1). Based on the findings in Harvest 1, an improved intervention was designed and deployed during Harvest 2 (2018–2019) [19, 24]. The enhanced RSH-S program during Harvest 2 included the following improvements: (i) increased rest time—for burned cane cutters, a regulated rest schedule was implemented during a 6-hour workday, with an additional 20 min of rest more evenly distributed throughout the day and for seed cutters, the workday remained 8 h but with two additional rest periods; (ii) improved access to shade and fluids—large shade tents and palatable fluids were placed close to workers (within 50 m) throughout the day; (iii) delayed cutting after burning—cutting was delayed for at least 12 h after a field was burned to reduce exposure to radiant heat and other pollutants; (iv) enhanced sanitation facilities—bathroom facilities were placed in the field, which is particularly necessary for women who otherwise tend to restrict their liquid intake to avoid urination, thereby increasing their risk for dehydration and heat-illness. The RSH-S program was further modified in Harvest 3 (2019–2020) to add breaks earlier in the day for the burned cane cutters and increase break periods for seed cutters. Assessment and improving organization management to optimize the implementation of the RSH-S program also became a focus in Harvest 3 [19, 24]. The COVID-19 pandemic occurred during Harvest 3, but sugarcane harvesting continued. The workers were required to wear facemasks, were specifically instructed to wash their hands before and after work shifts, were requested to keep physical distance, and were monitored for signs and symptoms of COVID-19, with testing provided by mill hospital staff as needed. Aside from modest improvements in the implementation, the RSH-S program was largely unchanged in Harvest 4 (2020–2021) and Harvest 5 (2021–2022).

ISA provided the research team with implementation and operational costs and worker outcomes data. These are data that ISA regularly collects to monitor production, employee turnover, absenteeism, and harvest-working days. ISA was blind to the purpose of providing these data to the research team. While the RSH-S intervention was designed to target those workers at the highest risk of occupational heat stress (e.g., burned cane cutters, seed cutters), the obtained data specifically included information related to the annual total costs of implementing the RSH-S program at ISA more broadly and were not specific to a given worker group. Costs included were specifically related to the production and distribution of an electrolyte solution (including ingredients and packaging, salaries for personnel, electricity consumption), equipment for new workers (e.g., hats, thermoses, shade tents, water reserves, etc.), worker training on occupational heat stress health risks and its prevention (i.e., a cost per worker), and salaries for personnel responsible for the implementation of the RSH-S intervention (e.g., health promotion employees). Fixed costs that were provided to the research team only one time from 2017 through 2022 were adjusted for inflation to estimate annual fixed costs. The research team was also provided with the total number of workers (including the number of burned cane cutters), burned cane cutter productivity measures (i.e., tons of sugarcane cut), absenteeism, and the number of AKI cases in burned cane cutters, including the cost for treatment at the mill’s on-site hospital. From the variable cost data, we calculated the cost savings due to the RSH-S program using data from 2017 (pre-RSH-S) as a baseline. Cost savings data focused specifically on burned cane cutters, given the ability to directly align burned cane cutter productivity with a monetary value (more information provided below). The use of ISA-wide costs relative to burned cane cutter-specific cost savings is an acknowledged limitation. However, this was deemed acceptable because we will be underestimating (and not overestimating) the cost savings. A summary of the data provided and used in the cost-benefit analysis has been included in Table 1. All amounts are presented in U.S. dollars.

**Table 1:** Variables employed in the cost-benefit and return on investment calculations.

FIXED RSH-S PROGRAM COSTS (ISA WIDE)	
Electrolyte beverage production	
Salaries for intervention staff	
Worker employment and training costs	
Worker equipment costs	
VARIABLE RSH-S PROGRAM OUTCOMES COSTS*	COST SAVINGS DUE TO RSH-S PROGRAM*
Cost of AKI treatment	Reduced need for AKI treatment
Cost of absenteeism (i.e., worker turnover)	Reduced absenteeism
Cost of productivity loss	Increased daily sugarcane cut

*Data specific to burned cane cutters only.

## Analyses

### Value of investments

#### Electrolyte beverage production

ISA provided a detailed cost estimate for their operations related to electrolyte beverage production and distribution. These costs can be broken down into three categories: ingredients and packaging ($140,462.36), operation salaries ($53,788.09), and energy usage (electricity) to produce and maintain the beverage ($32,294.43), which totaled $226,544.88 in 2020. These costs were subsequently adjusted for inflation to determine annual fixed costs from 2017 through 2022.

#### Equipment costs

Prior to 2017, subcontracting during the harvest period was regularly used at ISA, as in many other sugar mills in the region. In 2017, ISA eliminated subcontracting. With the formalization of the workforce and the establishment of an employer–employee working relationship, ISA invested in providing uniform equipment of specified quality for its cutters, some of which was important in implementing the RSH-S program. This equipment included making annual purchases of hats, shirts, shin guards, gloves, machetes, thermoses, water reserves, and shade structures. While there is no need to purchase new equipment for employees each harvest season, ISA estimates that these annual purchases could equip 180 workers per harvest to account for equipping new employees and replacing degraded equipment. ISA provided estimates on the total value of these purchases by category in 2022, amounting to $34,438.00. These costs were subsequently adjusted for inflation to determine annual fixed costs from 2017 through 2021.

#### Annual occupational safety and health training

The occupational health unit at ISA conducted training for the priority jobs in the RSH-S intervention (burned cane cutters, seed cutters) to review practices and ensure workers (and their supervisors) were adopting the RSH-S program appropriately to reduce the incidence of heat strain and associated illness [19]. The value of this training includes the dollar value of time dedicated per-harvest cycle and the materials produced to educate and refresh workers. In 2022, we estimate the cost per worker to be $7.21 at a total value of $2,617.23 (363 total burned cane cutters and seed cutters in 2022). These costs were subsequently adjusted for inflation to determine annual fixed costs from 2017 through 2021.

#### Health promotion staffing

A substantial portion of the intervention costs is attributed to ISA’s mill hospital employees dedicated to the promotion of RSH-S-related activities. We include the salaries for three health promotion officials totaling $51,308.65 in 2022. These costs were subsequently adjusted for inflation to determine annual fixed costs from 2017 through 2021.

### Value of benefits

#### Productivity

The aim of the RSH-S program was to improve occupational conditions for workers with the highest risk of occupational heat stress. After conducting initial rounds of monitoring and analysis to establish recommendations for ISA, the intervention was designed to primarily target two working groups: burned cane cutters and seed cane cutters. Given the ability to directly align burned cane cutter productivity (i.e., tons of sugarcane cut per worker per year) with a monetary value (see below), the productivity assessments in this case study only include burned cane cutters. We acknowledge that we are likely underestimating the potential productivity benefits of the RSH-S program, as, for example, our group has recently also identified improvements in seed cutters [18], not to mention other working groups where the quantification of productivity and subsequent financial implications is less straightforward. Although a limitation, we believe this is acceptable given that only including burned cane cutter productivity is an underestimate of the potential productivity benefits.

Using the data provided by ISA’s human resources department, we can estimate the total number of working days between 2017 and 2022 by multiplying the average number of workers per year by the total number of working days for burned cane cutters. Table 2 provides a breakdown of the average number of workers and total working days between 2017 and 2022 for the burned cane cutters and all employees.

**Table 2:** Average number of burned cane cutters and total number of workers at ISA, and total working days for burned cane cutters.

YEAR	TOTAL NUMBER OF BURNED CANE CUTTERS	TOTAL LABOR FORCE AT ISA	TOTAL NUMBER OF WORKING DAYS PER BURNED CANE CUTTER
2017	498	3775	143
2018	227	3503	136
2019	180	2755	124
2020	180	2527	101
2021	169	3028	123
2022	154	2932	156

The total number of burned cane cutters decreased throughout the observation period, while the total number of working days per burned cane cutter was relatively constant.

Using findings from our previous study conducted to estimate changes in production for burned cane cutters after RSH-S program implementation [18], measures of total potential production can be estimated in terms of cane tonnage harvested. Using this information, average yearly productivity can be calculated from the number of workers and the total number of working days for burned cane cutters. These data are presented in Table 3.

**Table 3:** Daily productivity for burned cane cutters from 2017 to 2022.

YEAR	AVERAGE SUGARCANE CUT PER WORKER PER DAY (IN TONS)
2017	5.7
2018	5.7
2019	5.6
2020	6.3
2021	5.9
2022	6.2

Daily productivity increased throughout the observation period.

**Figure 1:**
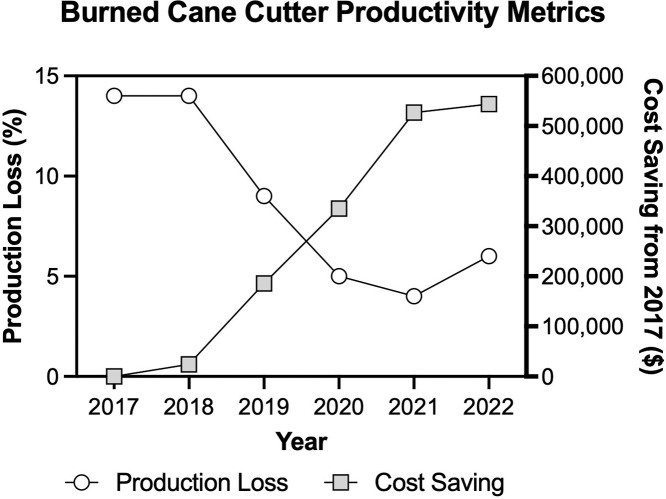
Calculated production losses and cost savings due to enhanced productivity from 2017 to 2022 in burned cane cutters. Production losses decreased and cost savings increased after initial implementation of the RSH-S program in Harvest 2 (2018–2019) and enhanced implementation in Harvest 3 (2019–2020).

To estimate the value of savings from increased production, we used absenteeism rates for burned cane cutters to calculate lost production based on potential productivity. ISA monitors the number of absenteeism days for workers across worker categories. Reasons for missing work are recorded in the following categories: unexcused absences, common illness, unpaid leave, and occupational risk. Table 4 provides a breakdown of the number of recorded working days absent by type of absence for the burned cane cutting crew. While the RSH-S program primarily focused on outcomes most closely associated with occupational heat stress, it is likely that the intervention improved working conditions more generally, which had an impact on other absenteeism categories such as unexcused absences and common illness. The logic behind this assumption is that if working conditions improve, workers will be more willing to maintain a regular working schedule in the presence of a safe and more welcoming working environment. Using the absenteeism figures provided by ISA, we calculated the total production loss due to missing workdays as the quotient of the total potential sugarcane harvest and lost production (Figure 1).

**Table 4:** Total workdays, missed workdays by reason, and total lost workdays for burned cane cutters.

YEAR	TOTAL WORKDAYS	UNEXCUSED ABSENCE	COMMON ILLNESS	UNPAID TIME OFF	OCCUPATIONAL RISK	TOTAL LOST WORKDAYS
2017	71,214	8723	1339	2	150	10,214
2018	30,872	2732	1260	87	143	4282
2019	22,320	843	930	185	104	2062
2020	18,180	585	56	179	37	857
2021	20,787	524	151	145	42	862
2022	24,024	923	314	209	25	1471

Total lost workdays generally decreased throughout the observation period.

It is conservatively estimated that one ton of harvested sugarcane produces 107 kg of raw sugar [25]. The average world market price for 1 kg of processed sugar in each year was determined as reported from the International Monetary Fund’s global price index [26]. From this information, the monetary value of improvements in total production losses (termed cost savings) can be calculated for each year relative to 2017, with the market price adjusted for inflation (Figure 1).

#### Health outcomes

**Figure 2:**
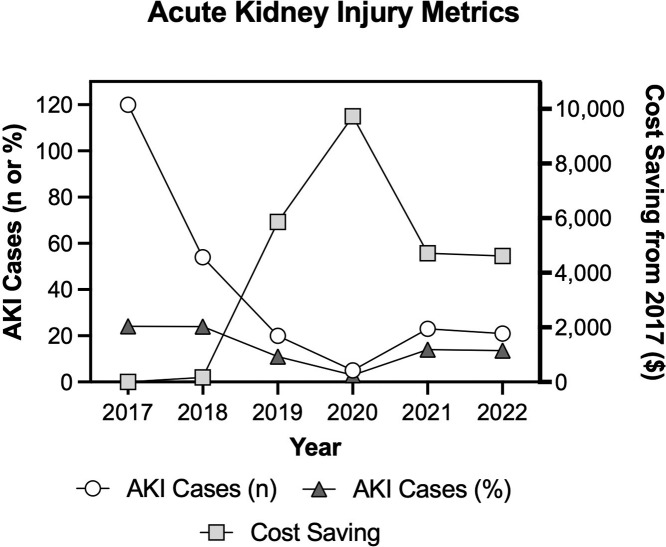
Total number of acute kidney injury (AKI) cases in burned cane cutters (*n*), as a function of the number of burned cane cutters (%) and the associated cost savings due to reduced AKI incidence in burned cane cutters. The number of AKI cases decreased, and cost savings increased after initial implementation of the RSH-S program in Harvest 2 (2018–2019) and enhanced implementation in Harvest 3 (2019–2020).

The primary adverse health outcome assessed was AKI. This assumes that AKI in this setting can be considered a heat-related illness [2]. Moreover, AKI is hypothesized to be central to the development of CKDnt [6]. The total number of AKI diagnoses in burned cane cutters decreased over time (Figure 2), from occurring in one out of every five workers in 2017 to one out of every 10 workers in 2022, as has been reported previously [15]. Thus, reductions in the occurrence of AKI can be expressed as savings to ISA. According to data provided by ISA in 2020, the total treatment cost for a worker diagnosed with AKI at the mill hospital was $253.48 and this value was adjusted for inflation for years 2017–2019 and 2021–2022. To capture savings associated with a reduction in costs for treating AKI, we took the per capita burned cane cutter AKI incidence among the working population at ISA in all years from 2017 to 2022. Cost savings were calculated as a function of the difference between treatment costs each year using the observed rate and what the observed rate might have been in the absence of the intervention (i.e., 2017), with the AKI treatment cost adjusted for inflation (Figure 2).

### Return on investment (ROI)

The dollar value of expected cost reductions was expressed as firm-level benefits that were then used to conduct an ROI assessment using the following standard ROI formula [27]:


ROI = (value of benefits – value of investments) / value of investments,

where the value of investments is the sum of the fixed costs, including those costs related to equipment, training, operations, and electrolyte beverage production, and the value of benefits is the sum of the cost savings from improvements in productivity and reductions in the incidence of AKI in burned cane cutters.

Given that the value of the benefits was calculated relative to 2017 (i.e., pre-RSH-S program implementation), an ROI could not be calculated for 2017. Therefore, ROI is presented as relative to that occurring in 2017.

## Findings

### Value of investments

Annual fixed costs for equipment, training, operations, and electrolyte beverage production are presented in Table 5.

**Table 5:** Annual fixed costs for implementation of the rest–shade–hydration–sanitation intervention.

YEAR	EQUIPMENT	TRAINING	OPERATIONS	ELECTROLYTE BEVERAGE PRODUCTION	TOTAL COSTS
2017	$28,927.92	$4,530.19	$43,098.72	$215,217.23	$291,774.05
2018	$29,616.68	$2,957.69	$44,124.88	$219,748.12	$296,447.36
2019	$29,961.06	$2,302.08	$44,637.96	$224,279.01	$301,180.11
2020	$30,305.44	$2,525.23	$45,151.04	$226,544.45	$304,526.16
2021	$32,027.34	$2,487.67	$47,716.44	$237,871.67	$320,103.12
2022	$34,438.00	$2,617.23	$51,308.00	$255,995.23	$344,358.46

The annual fixed costs were largely unchanged throughout the observation period.

### Value of benefits

Annual cost savings due to changes in production (i.e., improvements in productivity) and the treatment of AKI are presented in Table 6.

**Table 6:** Annual cost savings following implementation of the rest–shade–hydration–sanitation intervention in 2017.

YEAR	PRODUCTION SAVINGS	AKI TREATMENT SAVINGS	TOTAL SAVINGS
2017	—	—	—
2018	$24,027.35	$171.82	$24,199.17
2019	$185,868.50	$5,865.47	$191,733.97
2020	$335,070.43	$9,726.91	$344,797.34
2021	$526,825.50	$4,717.02	$531,542.52
2022	$543,823.21	$4,613.98	$548,437.19

Total savings, a function of production savings and AKI treatment savings, increased throughout the observation period.

### Return on investment

**Figure 3:**
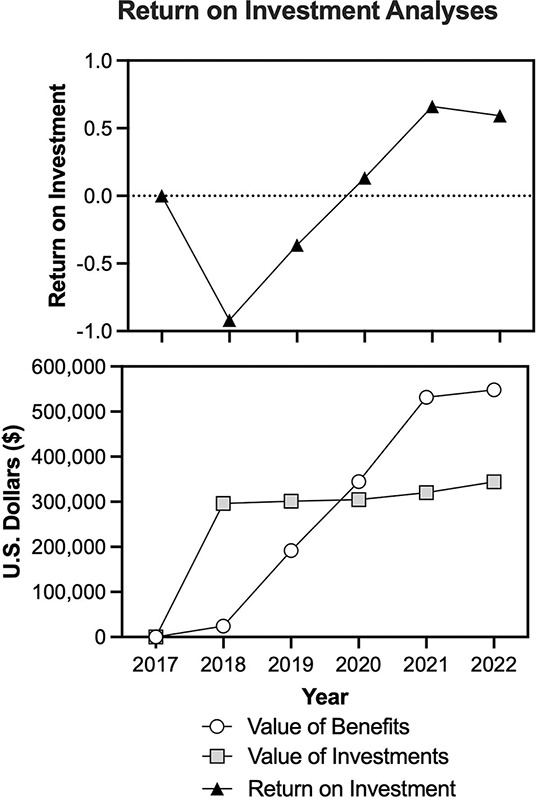
The value of the benefits and investments following the implementation of the rest–shade–hydration–sanitation program in 2018 (bottom) and quantification of the annual net return on investment (top). The costs of implementing the RSH-S program were offset by savings two years after initial implementation in Harvest 2 (2018–2019) and yielded a positive return on investment three years after implementation (Harvest 4, 2020–2021), which was sustained through 2022, the last year in which data were available.

The annual value of benefits, value of investments, and ROI for the implementation of the RSH-S program are presented in Figure 3. From 2020, there was a positive ROI, which escalated with improvements in RSH-S intervention implementation.

## Discussion

The purpose of this case study was to determine the economic impact of an RSH-S program implemented at the ISA sugar mill in Chichigalpa, Nicaragua. Chichigalpa has one of the highest known rates of CKDnt [8], which has been proposed to be predominantly caused by occupational heat stress-induced AKI [6, 28]. Implementation of the RSH-S program at ISA has already been shown to improve worker productivity [18], reduce the incidence of AKI [15], and attenuate cross-harvest reductions in kidney function [13, 14]. While preliminary evidence supports a beneficial economic impact of the implementation of an RSH-S program [19, 20], a comprehensive economic assessment of the potential financial benefits of an RSH-S program was previously lacking. To this end, our analysis demonstrates that as soon as three years following implementation of the RSH-S program the health and productivity benefits exceed the costs such that, for example, in 2022, for every $1.00 invested in the RSH-S program ISA saw a return of $1.60 (or a net return of $0.60) (Figure 3). Notably, even the average ROI over the five-year period was positive (0.02 or a return of $1.02 for every $1.00 invested), which takes into consideration the first two years of the intervention in which there was a negative ROI.

Given the vulnerability of outdoor workers to severe heat, which is being made worse by climate change, there is widespread acknowledgement of the need to provide RSH-S to outdoor workers to mitigate the short-term (e.g., heat illness, AKI) and longer term (e.g., CKDnt) health and safety implications of occupational heat stress [2]. However, a major obstacle for successful integration of RSH-S programs into the workplace is that both employers and workers worry that the reduced work time in the RSH-S program (i.e., increased rest breaks) will reduce workplace productivity and employee earnings, particularly when the work is piece-paid. Overall, the results presented in this case study suggest that the daily lost functional work time associated with RSH-S for the workers is offset by savings in (i) productivity, due to both reductions in absenteeism and increases in annual per-worker harvested tonnage and (ii) AKI treatment costs due to reductions in the incidence of AKI (Figure 2). Notably, despite implementation of the RSH-S program, which introduced additional rest resulting in a shorter overall time working, we observed modest improvements in burned cane cutter productivity (i.e., tons of sugarcane cut, Table 3, [18]). Given that burned cane cutters are piece-paid workers, this suggests that earnings are (at worst) not changed and that the workers are healthier (Figure 2). Thus, these findings indicate that there are financial and health benefits to both the employer and employee due to the implementation of the RSH-S program.

It is worth noting that the observed economic benefits of the RSH-S program are likely an underestimate, given the sole focus on burned cane cutters in the determination of cost savings. This was chosen due to the relative simplicity of linking burned cane cutter productivity (i.e., tons of sugarcane cut per worker per year) with a monetary value, the latter of which is a function of the yield of sugar per ton of sugarcane and the market price of sugar. We note that other groups of workers likely also displayed improvements in productivity. For example, Hansson et al. [18] recently reported an average of an 11% increase in productivity in seed cutters following implementation of the RSH-S program at this sugar mill. It is entirely possible that other working groups at ISA also experienced improvements in productivity. Notably, quantification of worker productivity is a challenge that is not exclusive to the sugarcane industry. Thus, future efforts should aim to improve the assessment and reporting of jobsite and worker productivity to better improve economic analyses before and after implementation of RSH-S programs.

Aside from initial preliminary assessments [19, 20], to our knowledge, a similar comprehensive economic assessment of the implementation of an RSH-S program has not been carried out. Thus, it is reasonable to consider whether the observed economic returns following the implementation of the RSH-S program are specific to ISA, a large sugar mill in Nicaragua, a lower middle-income country in Central America. It is well established that occupational heat stress reduces worker productivity [29] and increases the risk of heat illness, including AKI [30]. Thus, the observations made in this case study are likely transferable to other industries, job types, and locations where occupational heat stress is common. Nevertheless, it is impossible to ignore the potential that the economic effects of an RSH-S program may not be as profound in other climate regions where occupational heat stress is more seasonal (e.g., at higher or lower latitudes), for employers in more economically prosperous regions and in different industries, particularly those not heavily relying upon piece-paid work. Therefore, future research should examine the potential effects of RSH-S programs on both employee and employer economic returns in more diverse settings, as this information would be vital towards widespread adoption of RSH-S programs.

The focus of the current case study has been on the short-term economic implications of the implementation of an RSH-S program. That said, there may also be longer term societal benefits. For example, given the links between occupational heat stress, AKI, and CKDnt [6], it is likely that a proportion of those experiencing AKI will develop CKDnt. With its outsized impact on working-age men, CKDnt contributes to lost economic opportunity for households and communities [31]. There are also benefits to state-funded entities like the Nicaraguan social security and public health systems that provide pensions to sick workers. By reducing the incidence of AKI and CKDnt, the state can focus its efforts on addressing other public health issues. Therefore, ISA’s implementation of an RSH-S program can help mitigate these long-term negative societal effects while still maintaining a productive workforce.

Although clear benefits have been observed, it is important to note the limitations present in this case study. For example, almost all the data used in the analysis came from one source, introducing opportunities for reporting biases that should not be overlooked. Additionally, productivity was estimated using data related to worker totals, working days per activity, and estimates on daily worker production. A more accurate analysis would ideally include financial data directly related to tonnage harvested, sugar extraction yields, and revenue from those yields. There are also some other expenditures that were ultimately inaccessible to the research team, particularly related to other relevant health-related outcomes (e.g., injuries, other heat illnesses), which would potentially be positively impacted by the RSH-S program. Future studies should prioritize understanding data limitations and establishing protocols for data collection and sharing to improve understanding of the intervention impact. A final limitation is that this case study simply compares the before- and aftereffects of the RSH-S program. Thus, more complex designs (e.g., those employing randomization, addressing certain confounders, including control groups, etc.), including multiple job sites and industries, if found feasible, are needed to better infer that the RSH-S program caused the observed economic changes. That said, it should not be ignored that this case study provides a firm foundation from which future work can be carried out.

## Conclusion

The RSH-S program at ISA, a sugar mill in Nicaragua, yielded health and productivity benefits that ultimately resulted in large economic returns five years after implementation. The program’s success in reducing AKI and increasing productivity highlights the strategic importance of creating safer working conditions, particularly when under occupational heat stress conditions. The positive ROI for this intervention after only three years indicates that an RSH-S program can likely be applied across the sugarcane industry and perhaps in other industries where occupational heat stress is common, although further research is needed. Future research should also aim to expand this cost-benefit analysis to identify societal returns, and, in the context of publicly funded programs, taxpayer returns for occupational safety and health interventions.

## Data Availability

Data will be made available upon reasonable request to the corresponding author.
